# Phenotypic and genomic characterization of *Castellaniella ginsengisoli*, an emerging pathogen associated with disease in birds

**DOI:** 10.1128/spectrum.03197-25

**Published:** 2026-02-23

**Authors:** Yi-Chen Luo, Tiffani Allen, Jenny Nicholds, Mary Ard, Tatum D. Mortimer, Grazieli Maboni

**Affiliations:** 1 Athens Veterinary Diagnostic Laboratory, College of Veterinary Medicine, University of Georgia 1355, Athens, Georgia, USA; 2 Best Dressed Chicken, Ward, South Carolina, USA; 3 Prairie Livestock Veterinarians, Red Deer, Canada; 4 Georgia Electron Microscopy, University of Georgia 1355, Athens, Georgia, USA; 5 Department of Population Health, College of Veterinary Medicine, University of Georgia 1355, Athens, Georgia, USA; JMI Laboratories, North Liberty, Iowa, USA

**Keywords:** antimicrobial susceptibility testing, minimum inhibitory concentration, broiler breeders, whole-genome sequencing, pathogenicity, phenotypic characterization, *Castellaniella*

## Abstract

The environmental bacterium *Castellaniella* revealed its clinical relevance from case reports, causing fatal infections in animals. The lack of phenotypic and genomic characterization may have historically contributed to the scattered identification of *Castellaniella* in the field and in the laboratory. This pioneer study addressed this knowledge gap by performing phenotypic and genomic characterization of *C. ginsengisoli* isolated from 22 clinical cases of chickens. We observed that *C. ginsengisoli* isolates exhibited three colony morphologies on blood agar and presented flagella under transmission electron microscopy (TEM) examination. Antimicrobial susceptibility testing showed high minimum inhibitory concentrations (MICs) for penicillins, indicating a potential intrinsic mechanism of resistance to ß-lactams. Efflux pump genes (*adeF, qacG*) and β-lactamase genes were identified in the whole genomes of all isolates, which could be linked to the high MICs observed for β-lactams. Low MICs for sulfonamides and tetracyclines, among other antimicrobials, were observed for most of the isolates, suggesting a potential treatment option for birds and other affected animals. We further confirmed that all isolates belong to the species *C. ginsengisoli* through the average nucleotide identity analysis. Additional genomic analysis revealed several putative virulence genes, including superoxide dismutase (*sodB*), adhesin (*tadB*), outer membrane (*ompA*), iron scavenging gene (*fur*), and secretion systems. This study documented, for the first time, the phenotypic features, *in vitro* activity of antimicrobials, and provided a comprehensive genomic characterization of clinical isolates of *C. ginsengisoli*.

**IMPORTANCE:**

The perception of *Castellaniella ginsengisoli* as an environmental bacterium has led to a lack of specific diagnostic tools, hindering the characterization of clinically associated isolates. With the increasing number of cases of *Castellaniella* in birds, there is a need to define characteristics to assist identification, therapeutic guidance, and understanding its potential pathogenicity mechanism. This study provided a comprehensive characterization of 22 *C. ginsengisoli* clinical isolates from chickens, which included colony morphology features, the *in vitro* activity of antimicrobials, and the resistant genetic markers identified through genomic characterization. We further provided a list of putative virulence genes and suggested how these genes may participate in the process of pathogenicity. This novel, comprehensive study laid the foundation for future research into *C. ginsengisoli,* which is likely an emerging pathogen in chickens as well as other animals.

## INTRODUCTION

The genus *Castellaniella* comprises gram-negative, facultatively anaerobic, motile, catalase-positive, and oxidase-positive rods belonging to the family *Alcaligenaceae*, typically recovered from soil and aquatic environments (1–3). Previous research on this metabolically versatile genus has primarily focused on its potential applications in bioremediation (4–10). However, sporadic reports have associated *Castellaniella* with fatal disease in animals. The earliest account, in 2011, described infection in a harbor porpoise (*Phocoena phocoena*) presenting with chronic orchitis and epididymitis (11). Clinical relevance increased with a subsequent report of an outbreak in which more than 10 Daurian pikas (*Ochotona daurica*) died with suppurative inflammation and abscesses in multiple organs (12). More recently, our group documented 20 cases of *Castellaniella* infection in chickens over a 5-year period, characterized by swollen wattles containing caseous exudate, lameness with hock synovitis, and elevated mortality (13).

The clinical manifestations of *Castellaniella*-associated disease in chickens resemble those of fowl cholera caused by *Pasteurella multocida* (13). Given this similarity, veterinarians may have empirically treated affected flocks with β-lactams, the first-line therapy for *P. multocida*. In contrast, while *P. multocida* is generally susceptible to β-lactams (14, 15), members of the *Alcaligenaceae* family typically exhibit intrinsic resistance to this drug class (16–19). Notably, the *in vitro* antimicrobial susceptibility profile of *Castellaniella* has never been systematically evaluated, limiting evidence-based treatment recommendations. Furthermore, although individual case reports suggest pathogenic potential, the lack of comprehensive evidence has hindered understanding of its virulence mechanisms. In addition, due to the absence of detailed phenotypic and genomic characterization and a major diagnostic gap, *Castellaniella* infections in birds and other animal species may have been historically underdiagnosed in the field and diagnostic laboratories.

To address these knowledge gaps, we conducted a comprehensive phenotypic and genomic analysis of *Castellaniella* isolates recovered from post-mortem lesions in chickens. The specific objectives of this study were to (i) characterize colony morphology and biochemical traits to facilitate diagnostic identification, (ii) determine minimum inhibitory concentrations (MICs) of antimicrobials to guide therapeutic decision-making, and (iii) analyze whole-genome sequences to confirm species taxonomy and identify putative antimicrobial resistance and virulence determinants. To our knowledge, this represents the first integrated characterization of *Castellaniella* clinical isolates.

## MATERIALS AND METHODS

### Isolate collection

Diagnostic samples were collected from commercial chickens in the United States between March 2018 and March 2024. Swabs from post-mortem lesions, including swollen hock joints or swollen wattles, were submitted for aerobic culture on 5% sheep blood agar and Luria-Bertani (LB) agar at 37℃ for 24–48 h. This collection included 20 isolates previously described in a clinical case series (13) and two additional isolates recovered in 2024. Bacterial colony morphology was examined based on the following characteristics: size, color, surface, shape, and margins. To determine the optimal growth temperature, purified isolates were subsequently cultured on 5% sheep blood agar and incubated at 25°C, 30°C, 37°C, and 42°C. The bacterial taxonomy was identified to genus level by Sanger sequencing the 16S rRNA gene PCR products, which were approximately 1,500 base pairs in size (20). Sequences were compared to the National Center for Biotechnology Information (NCBI) database. Isolates that belong to the *Castellaniella* genus were included in this study.

### Antimicrobial susceptibility testing

Antimicrobial susceptibility testing (AST) was performed to establish the minimum inhibitory concentration (MIC) of antimicrobials using the Sensititre Avian AVIAN1F Vet AST Plate (Thermo Fisher Scientific, Waltham, MA). Bacterial colonies were suspended in sterile water to achieve a 0.5 McFarland turbidity standard. Subsequently, 10 µL of this suspension was inoculated into 10 mL of Mueller-Hinton Broth (MHB) to prepare the final inoculum, and 50 µL of the final inoculum was dispensed into each well of the plate. To evaluate growth characteristics, the assay was performed using both standard MHB and MHB supplemented with horse blood. The following antimicrobials were included on the plate: amoxicillin, ceftiofur, clindamycin, enrofloxacin, erythromycin, florfenicol, gentamicin, neomycin, novobiocin, oxytetracycline, penicillin, spectinomycin, streptomycin, sulfathiazole, sulfadimethoxine, tetracycline, trimethoprim-sulfamethoxazole, and tylosin tartrate. The Sensititre plates were incubated at 34°C–36°C for 24 h and analyzed using Biomic V3 Microbiology system (Giles Scientific, Santa Barbara, CA). In addition, Vitek 2 AST-GN96 and Epsilometer testing (E-test) for amoxicillin and penicillin (bioMérieux, Marcy l'Etoile, France) was performed according to the manufacturer’s instructions to test for the presence of extended-spectrum beta-lactamase and provide extended MIC information.

### Biochemical analysis

Biochemical analysis was performed using the VITEK 2 Gram Negative (GN) ID Card (bioMérieux, Marcy-l'Étoile, France) following the manufacturer’s instructions. Results were interpreted using the ID-GNB database of the VITEK 2 system. Kinetic fluorescence measurements were taken every 15 min over a 3-h incubation period. The GN ID system included 47 biochemical tests to assess carbon source utilization, enzymatic activities, and antibiotic resistance, aiding in bacterial identification.

### Phylogenetic analysis and pan-genome analysis

Genome sequences of isolates from chicken lesions, as previously described in our genome announcement manuscript (21), were used as query sequences to confirm their species-level taxonomy. The reference sequence database was compiled using available *Castellaniella* spp. genomes from the NCBI RefSeq database (Table S1). FastANI (v1.33) (22) was used to calculate the average nucleotide identity (ANI) through pairwise comparisons between the query and the reference genome. The pan-genome analysis utilized Panaroo (v1.3.2) (23) for investigation, Gubbins (v3.3.5) for core-gene tree construction (24), Phandango for visualization (25), and Panstripe (v.0.3.1) (26) for analyzing gene gain and loss rates. Additionally, an in-house R script was used to simulate pangenome accumulation, generating a graph to visualize gene gain and loss across different sampling sizes.

### Identification of antimicrobial-resistance genes and virulence genes

We incorporated three different databases for antimicrobial-resistance (AMR) gene detection with default settings: (i) AMRFinderPlus (v.3.11.18) with database version 2023-9-26.1 (27); (ii) Resfinder (v4.5.0), with database version 2.3.2 (28), which was used to search for point mutations and acquired resistance genes. The species was specified as “Other” and a minimum coverage of 60% and a threshold identity of 90% were applied; and (iii) the Comprehensive Antibiotic Resistance Database (CARD v.6.0.3, database version 3.2.9) (29), with the “Perfect” algorithm applied to detect the exact matches to the curated reference database, and the “Strict” algorithm to identify previously unknown variants of known AMR genes. Furthermore, Bakta (30) was used for genome annotation, and the resulting annotation files were screened for the keyword “lactamase” to search for potential AMR mechanisms.

To elucidate the potential pathogenic mechanisms of these isolates, we sought to identify a comprehensive set of putative virulence factors covering the entire pathogenic process, including colonization, nutrient acquisition, immune evasion, and the ultimate damage (31). We combined two approaches for putative virulence gene detection. First, we submitted our sequences to the Virulence Factor Database (VFDB) interactive web interface, which identified virulence factors by performing both conventional sequence similarity comparisons and homolog and ortholog identifications against the VFDB data set (32). Because none of the *Castellaniella* species were included in the VFDB data set, genes with functions similar to known virulence factors might be missed due to database limitations. To account for the limitation, a manual keyword search was performed on the Bakta annotation results. This curated list of search terms, compiled from the VFDB and from virulence genes documented in *P. multocida* and *Bordetella* spp. of the *Alcaligenaceae* family (33–36), included both generic keywords and gene name prefixes to screen for entire virulence gene clusters (Table S2). For each included putative virulence gene, its identification was verified either by its annotated product name or by confirming its predicted function through UniProt, KEGG, or relevant publications. All identified putative virulence genes were categorized into four panels based on their potential roles in disease development as follows: (i) signaling/regulation, (ii) evasion/damage, (iii) ion and nutrient acquisition/homeostasis, and (iv) motility/adherence. Briefly, genes related to secretion systems (types I, II, III, IV, and VI), efflux pump transporters, redox transport systems, and regulatory systems were assigned to the signaling/regulation category, reflecting their likely involvement in quorum sensing and the modulation of host cell signaling. The evasion/damage panel comprised genes associated with biofilm formation, capsule biosynthesis, nitrate/nitrite reduction, oxidative stress resistance, O-antigen regulation, and toxin production. Genes involved in the scavenging of ions and nutrients, as well as the maintenance of cellular homeostasis, were grouped under the ion and nutrient acquisition/homeostasis panel. Finally, the motility/adherence panel included genes that regulate the formation of adhesins, fimbriae, pili, and flagella, as well as those involved in chemotaxis.

### Transmission electron microscopy   

* Castellaniella* suspension cultures were processed for routine transmission electron microscopy (TEM) at the Georgia Electron Microscopy facility, University of Georgia. Bacterial suspensions were submitted in a fixative of 2% paraformaldehyde, 2% glutaraldehyde in 0.1M Cacodylate-HCl buffer, pH 7.25. The samples were washed several times in 0.1M Cacodylate-HCl buffer, pH 7.2, with light centrifugation before agar-enrobing each sample in 3% aqueous Noble Agar (60°C). Once the agar-bacteria pellets were set, they were placed back in buffer before post-fixation in 1% osmium tetroxide in 0.1M Cacodylate-HCl buffer, pH 7.2, for 1 h. The pellets were washed several times in deionized water and then dehydrated in an ethanol series (30%, 50%, 75%, 95%, 95%, 100%, 100%), then cleared in two quick changes each of acetone and then propylene oxide. The sample pellets were infiltrated with 2:1, 1:1, and 1:2 mixtures of propylene oxide and Mollenhauer’s Epon-Araldite plastic mixture (37): 2 h for the 2:1 mixture, 2 h for the 1:1 mixture, and overnight in the 1:2 mixture. The embedded samples were polymerized in a 70°C–80°C oven overnight. The 100% Epon-Araldite mixture was exchanged out twice: one for 3 h, and then for 2 h before embedding the pellets in flat embedding molds (38). During ultramicrotomy, 60–70 nm sections were obtained and placed on 200-mesh copper grids. Sections were post-stained with 2% aqueous uranyl acetate and Reynolds lead citrate (39) prior to viewing with a JEOL JEM-2100PLUS transmission electron microscope (JEOL Ltd., Tokyo, Japan) operating at 120 kV of accelerating voltage. Images were acquired using an AMT NanoSprint15L-MarkII High Sensitivity Scientific Complementary Metal-Oxide-Semiconductor (sCMOS) TEM Camera (Advanced Microscopy Techniques, Woburn, MA, USA) with a resolution of 5,056 × 2,960 pixels. Colors were added using Adobe Photoshop 2025 to enhance the structure of the cell wall.

Part of the fixed bacterial cultures was selected for the negative staining technique. This technique was performed as follows: 10–15 μL of sample was placed on a 300-mesh formvar, carbon-coated grid (previously glow-discharged) for 15 min. The excess was removed using filter paper and stained with 2% phosphotungstate for 15 s and allowed to dry before viewing with the JEM2100Plus TEM at 120 kV accelerating voltage.

## RESULTS

### Growth preference, morphological and biochemical characterization

**Fig 1:**
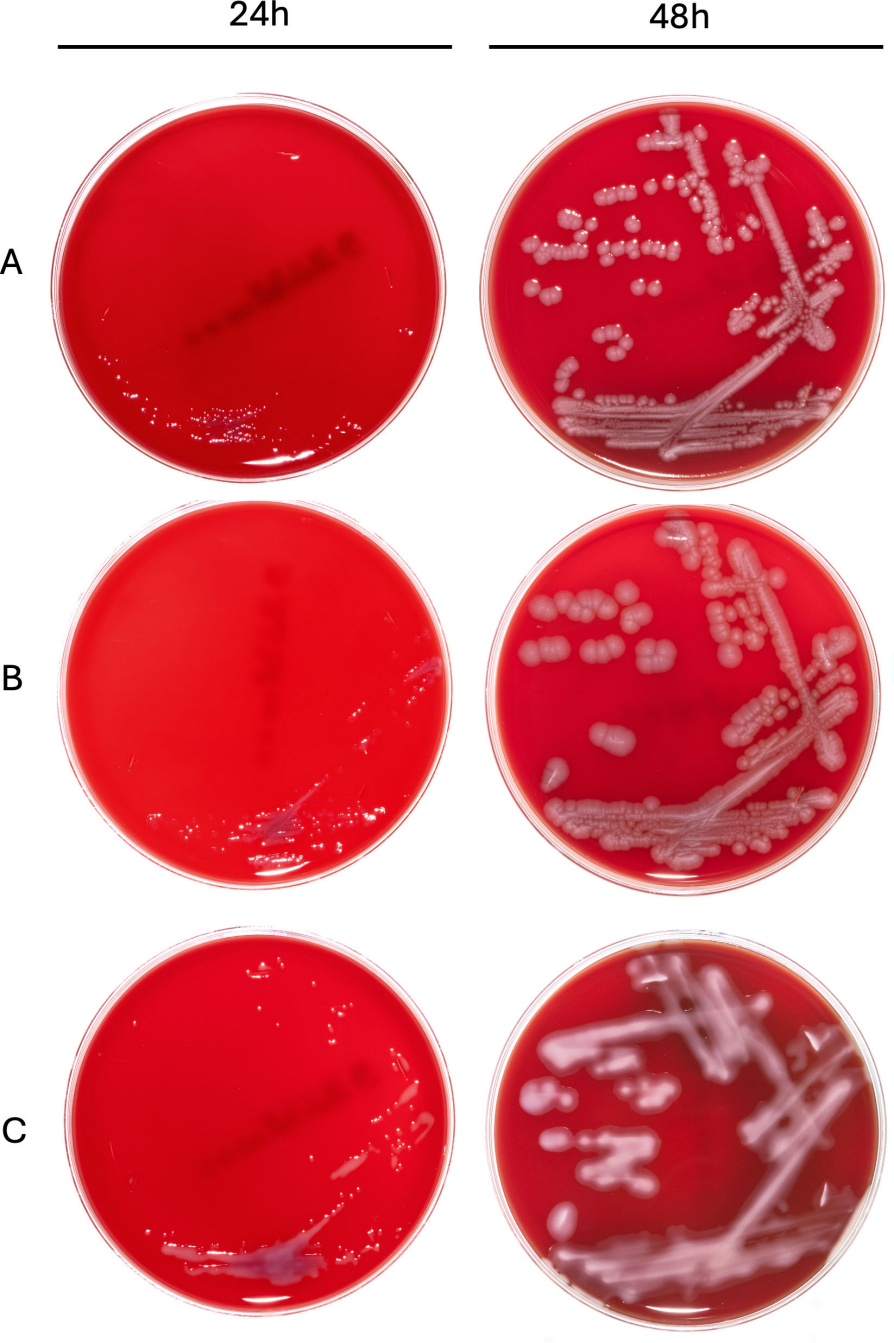
Different isolates of *C. ginsengisoli* on blood agar present three distinct colony morphologies. After 24 h, colonies from the three forms were uniformly small (approximately 1 mm), non-hemolytic, circular, flat, smooth, and transparent. However, after a 48-h incubation, the following distinct forms from different isolates became apparent: (**A**) Non-mucoid discrete colonies with a smooth, glistening surface and an entire margin (27% of isolates). (**B**) Non-mucoid discrete colonies with a smooth, less glistening surface and a curled margin (9% of isolates). (**C**) Mucoid colonies coalesced into continuous growth, with a smooth, glistening surface and a slightly transparent matrix at the edges (64% of isolates).

**Fig 2:**
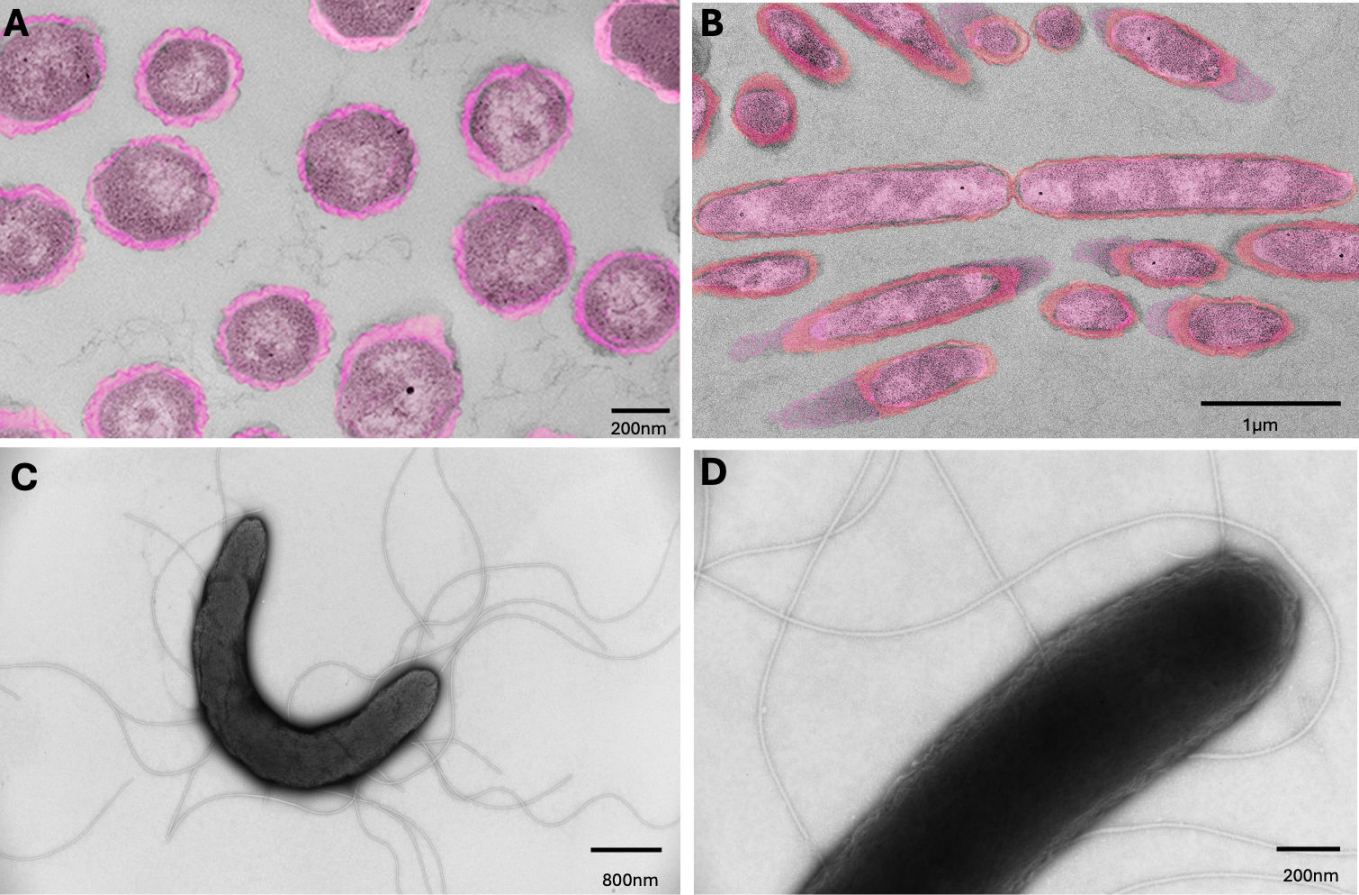
Flagella of *C. ginsengisoli* visualized under TEM. Bacteria were cultured on Luria-Bertani broth for 24 h prior to the examination. (**A**) Transverse plane section of *C. ginsengisoli*. (**B**) Sagittal plane section of *C. ginsengisoli*. Colors were added to (**A**) and (**B**) using Adobe Photoshop 2025 to enhance the ultrastructure of the bacteria. (**C**) Negative staining showed the presence of flagella. (**D**) Negative staining showed no fimbriae and pili on the bacterial surface.

Our results indicated that *C. ginsengisoli* grew at all tested temperatures (25°C, 30°C, 37°C, and 42°C), with optimal growth occurring at either 37°C or 42°C. On blood agar, *C. ginsengisoli* formed small (approximately 1  mm), non-hemolytic, circular, flat, smooth, and transparent colonies after 24 h of incubation. Between 24 and 48 h, the colonies grew larger (around 3 mm) and developed a circular, smooth, white appearance. Morphology varied slightly between the 22 isolates. In non-mucoid isolates, colonies remained discrete, with a smooth, glistening surface and an entire margin (Fig. 1A); this morphology was observed in 27% (6/22) of the isolates. Another non-mucoid morphology formed a less glistening surface with a slightly curled margin (Fig. 1B), accounting for 9% (2/22) of the isolates. In contrast, mucoid isolates tended to coalesce into a continuous growth, exhibiting a smooth, glistening surface and a slightly transparent matrix at the edges of the main white colony bodies (Fig. 1C). The mucoid morphology represents the majority, comprising 64% (14/22) of the isolates. Under TEM, the bacteria were approximately 400 nm in diameter and 2.5 µm in length (Fig. 2A and B). Flagella were observed on the negative stain (Fig. 2C and D), while no fimbriae, pili, and capsule structures were observed with the current sample processing technique.

For carbon source utilization, all *C. ginsengisoli* isolates were positive for L-lactate alkalinization, while most were positive for L-lactate assimilation, succinate alkalization, L-malate assimilation, and the Ellman test. A few isolates utilized citrate and histidine, but none of the 22 *C. ginsengisoli* isolates utilized the other tested carbon sources (Fig. S1A). Among the tested enzymes, all isolates showed consistent L-proline arylamidase activity (100% positive), and most isolates were positive for tyrosine arylamidase, while other tested enzymes were predominantly negative (Fig. S1B). For diagnostic purposes, the biochemical profile for *C. ginsengisoli* was summarized in Fig. S1C using standard VITEK 2 interpretive ranges.

### Antimicrobial susceptibility profile and AST testing methods

All *C. ginsengisoli* isolates showed low MICs for amphenicol (florfenicol) and aminoglycosides (gentamicin and neomycin) (Table 1). Conversely, all isolates exhibited MIC values exceeding the highest concentration tested for beta-lactams (ceftiofur, penicillin, amoxicillin), lincosamide (clindamycin), macrolides (erythromycin, tylosin), and aminocoumarin (novobiocin) (Table 1). For other drugs, including aminoglycoside (streptomycin), aminocyclitol (spectinomycin), fluoroquinolones (enrofloxacin), sulfonamides (sulphadimethoxime, sulphathiazole, sulfamethoxazole trimethoprim), and tetracyclines (tetracycline and oxytetracycline), the MICs mostly fell within or below the tested range. However, a few isolates in this group showed MIC values exceeding the testing limit for aminocyclitol (spectinomycin) and sulfonamides (sulphadimethoxime, sulphathiazole, sulfamethoxazole, and trimethoprim).

**TABLE 1:** The minimum inhibitory concentration of antimicrobials against *C. ginsengisoli* clinical isolates*^c^*^,^*^d^*

		Number of isolates with MIC (µg/mL) value^*a*^													
Drug class	Drug	0.12	0.25	0.5	1	2	4	8	16	20	32	64	128	256	512
Amphenicol	Florfenicol				22										
Aminoglycoside	Gentamicin			22											
	Streptomycin							19	1		1		1		
	Neomycin					22									
Aminocoumarin	Novobiocin							22^*^							
Aminocyclitol	Spectinomycin							11	9				2*		
ß-lactams	Amoxicillin									22^*^					
	Penicillin G								22^*^						
	Ceftiofur							22^*^							
Fluoroquinolones	Enrofloxacin	15	6	1											
Lincosamides	Clindamycin							22^*^							
Macrolides	Erythromycin							22^*^							
	Tylosin										22^*^				
Sulfonamides	SDM										20			1	1^*^
	Sulfathiazole										20				2^*^
	TMS^*b*^			20			2^*^								
Tetracyclines	OXT					2	18	2							
	Tetracycline					17	5								

^*a*^ Test results obtained from Avian AVIAN1F Vet AST plate. The white areas represented the tested range of an antimicrobial agent, while the grey areas represented the concentration not tested. The numbers indicated the number of isolates exhibiting MIC values equal to or lower than the listed number, while the asterisked numbers (*) within the gray areas indicated the number of isolates exhibiting MIC values higher than the testing range.

^*b*^ Data represent the concentration of trimethoprim.

^*c*^ TMS, trimethoprim-sulfamethoxazole; SDM, sulfadimethoxime; OXT, oxytetracycline; MIC, minimum inhibitory concentration.

^*d*^ Total number of isolates: 22.

The high MICs to β-lactam were confirmed by E-test strips, and all isolates exhibited MICs exceeding the E-test tested concentrations (up to 32 µg/mL for penicillin and 256 µg/mL for amoxicillin) (Table S3). Although the Vitek 2 GN96 card did not identify an extended-spectrum β-lactamase (ESBL) in *C. ginsengisoli*, the amoxicillin/clavulanic MICs were significantly lower (4–8 µg/mL) compared to the high MICs for amoxicillin alone (>256 µg/mL) (Table S3). 

The MIC results showed only minor differences when comparing Mueller-Hinton broth (MHB) with and without horse blood for isolates yielding values within the testing limits. Although some variations were observed, differences were typically limited to one to two dilution steps (Table S4). However, as a significant proportion of MIC values fell outside the testing ranges, a direct quantitative comparison was not possible for those antimicrobials. Therefore, conclusions regarding the interchangeability of these two methods are limited by the range of the current testing panel.

### Species identification and pangenome

**Fig 3:**
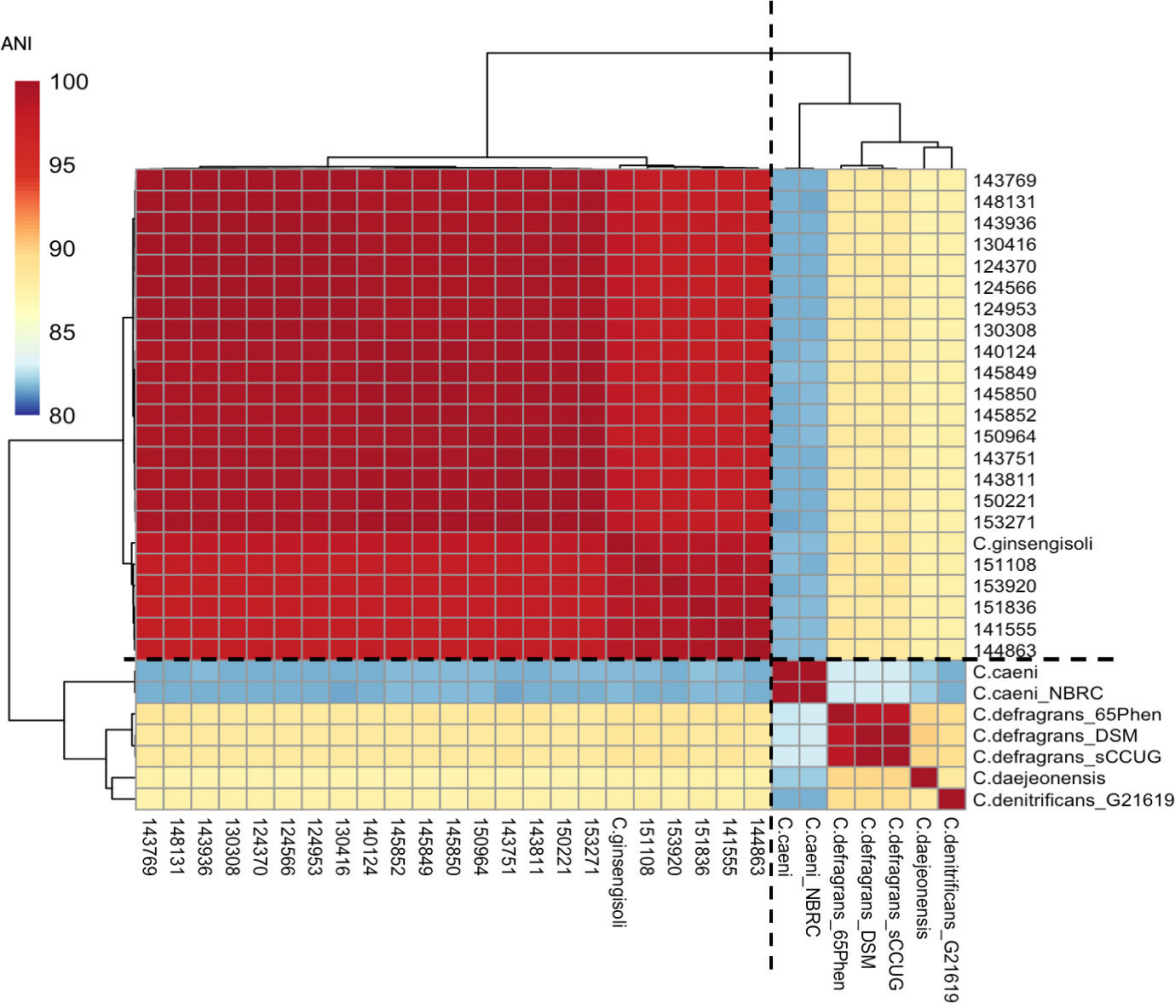
ANI heatmap confirmed 22 clinical isolates as *C. ginsengisoli*. The analysis was performed using FastANI (v1.33), which included eight *Castellaniella* spp. reference genomes from NCBI RefSeq and 22 clinical *Castellaniella* isolates. The heatmap displayed ANI values between all genome pairs, clustering based on similarities (dashed lines indicated the two major clusters). Results confirmed all the clinical *Castellaniella* isolates clustered with *C. ginsengisoli* (ANI > 95%).

We sequenced the 22 complete genomes of *C. ginsengisoli* isolated from lesions of chickens (21). The basic characteristics of these NCBI-available genomes are summarized in Table 2. The genome sizes range from 2.88 to 3.03 Mb, with GC content varying between 65.9% and 66.4%. The number of coding sequences (CDS) in these isolates spans from 2,631 to 2,812 based on Bakta annotation (30). Comparison of our *Castellaniella* sequences with other *Castellaniella* spp. (Listed in Table S1) showed ANI values exceeding 98% with *C. ginsengisoli*. In contrast, ANI values against other *Castellaniella* reference sequences were below 90% (Fig. 3). Since an ANI of ≥95% was the suggested threshold for prokaryotic species circumscription (40), our findings confirmed that all our isolates belong to *C. ginsengisoli*.

**TABLE 2:** Accession numbers and genome features of *C. ginsengisoli* isolated from chicken lesions^*a*^

Isolate	Collection month	Clinical specimen	Genome size (bp)	CDS	GC content (%)	Accession number
124370	2018-03	Hock joint	2,948,785	2733	65.9	CP158273
124566	2018-04	Hock joint	2,948,846	2731	65.9	CP158272
124953	2018-04	Hock joint	2,948,849	2733	65.9	CP158271
130308	2019-06	Hock joint	2,948,845	2734	65.9	CP158270
130416	2019-06	Hock joint	2,948,848	2735	65.9	CP158269
140124	2021-06	Hock joint	2,937,069	2719	65.9	CP158268
141555	2021-09	Wattle	2,951,780	2686	66.1	CP158267
143751	2022-02	Wattle	2,880,196	2631	66.4	CP158266
143769	2022-02	Hock joint	2,958,439	2756	65.9	CP158265
143811	2022-03	Hock and wattle	2,880,193	2633	66.4	CP158264
143936	2022-03	Hock and wattle	2,911,400	2683	65.9	CP158263
144863	2022-05	Wattle	3,021,650	2785	65.9	CP158262
145849	2022-08	Wattle	2,939,577	2724	65.9	CP158261
145850	2022-08	Wattle	2,939,560	2723	65.9	CP158260
145852	2022-08	Wattle	2,939,522	2734	65.9	CP158259
148131	2023-02	Hock	2,958,440	2757	65.9	CP158258
150221	2023-06	Hock	2,937,616	2730	65.9	CP158257
150964	2023-08	Wattle	2,931,857	2659	65.9	CP158256
151108	2023-09	Wattle	2,948,848	2711	66	CP158255
151836	2023-11	Wattle	3,032,368	2812	66	CP158254
153271	2024-02	Hock	2,939,711	2730	65.9	CP158253
153920	2024-03	Hock	3,014,323	2724	66.1	CP158252

^*a*^ CDS, coding sequences.

**Fig 4:**
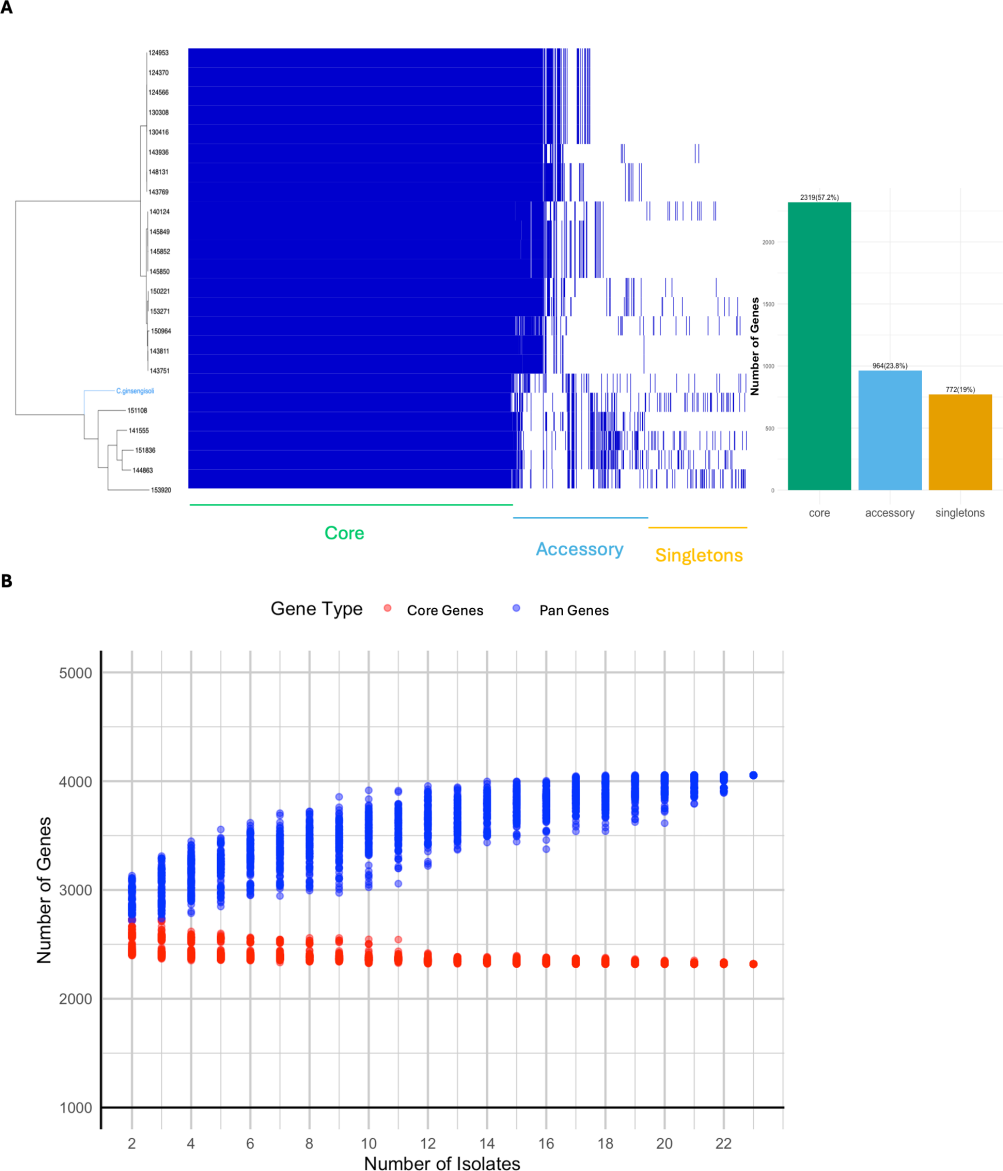
Pangenome analysis of *C. ginsengisoli* clinical isolates. Pangenome analysis was performed using Panaroo, while core-gene tree construction was conducted with Gubbins and visualized using Phandango. (**A**) The bar chart shows the proportion of the pangenome classified as core (present in all isolates), accessory (present in 5%–99% of isolates), and singletons (present in less than 5% of isolates). (**B**) The cumulative curve was generated using a permutation-based method by sampling 23 isolate genomes without replacement (with *n* = 2–23), simulating the sequential addition of one genome at a time. For each *n*, the permutations were repeated with 100 iterations to avoid bias. The 23 genomes included one *C. ginsengisoli* reference genome (Accession No: GCF_039523235.1) and 22 clinically associated *C. ginsengisoli* genomes.

We included the RefSeq *C. ginsengisoli* genome, along with our 22 *C. ginsengisoli* isolate sequences, in a pangenome analysis. There were 2,319 core genes (57.2%) defined as genes present in all genomes, and 1,736 accessory genes (42.8%). The accessory genes comprised 964 genes (23.8%) that were found in more than two genomes (between 5% and 99% of the genome population), and 772 genes (19%) that were present in only one genome (Fig. 4A). Altogether, these genes formed the pan-genome of *C. ginsengisoli*, consisting of 4,055 genes. The accumulation graph indicated a closed pan-genome, as the pan-genome curve gradually approached a plateau with the addition of more genomes. Meanwhile, the number of core genes stabilized, showing minimal reduction as more genomes were included (Fig. 4B). Our statistical analysis using Panstripe (26) further supported this view, showing an insignificant association between branch lengths and the number of gene gain and loss events (*P*-value > 0.05), indicating a closed pangenome for the 23 *C. ginsengisoli* sequences.

### AMR genes and virulence genes identification

We detected *sul2* in two isolates (144863 and 151836), *aadA7* in two other isolates (141555 and 151836), and *aph* (3″)-lb and *aph* (6)-ld in one isolate (150964), using the AMRFinderPlus, Resfinder, and CARD databases. The CARD database identified two additional efflux pump genes, *adeF* and *qacG*, in all 22 isolates. We further manually searched for resistance markers within the genome annotation files, and we found two β-lactamase genes, *bla* (class A β-lactamase) and a β-lactamase gene with no specified gene name (Table S5).

**Fig 5:**
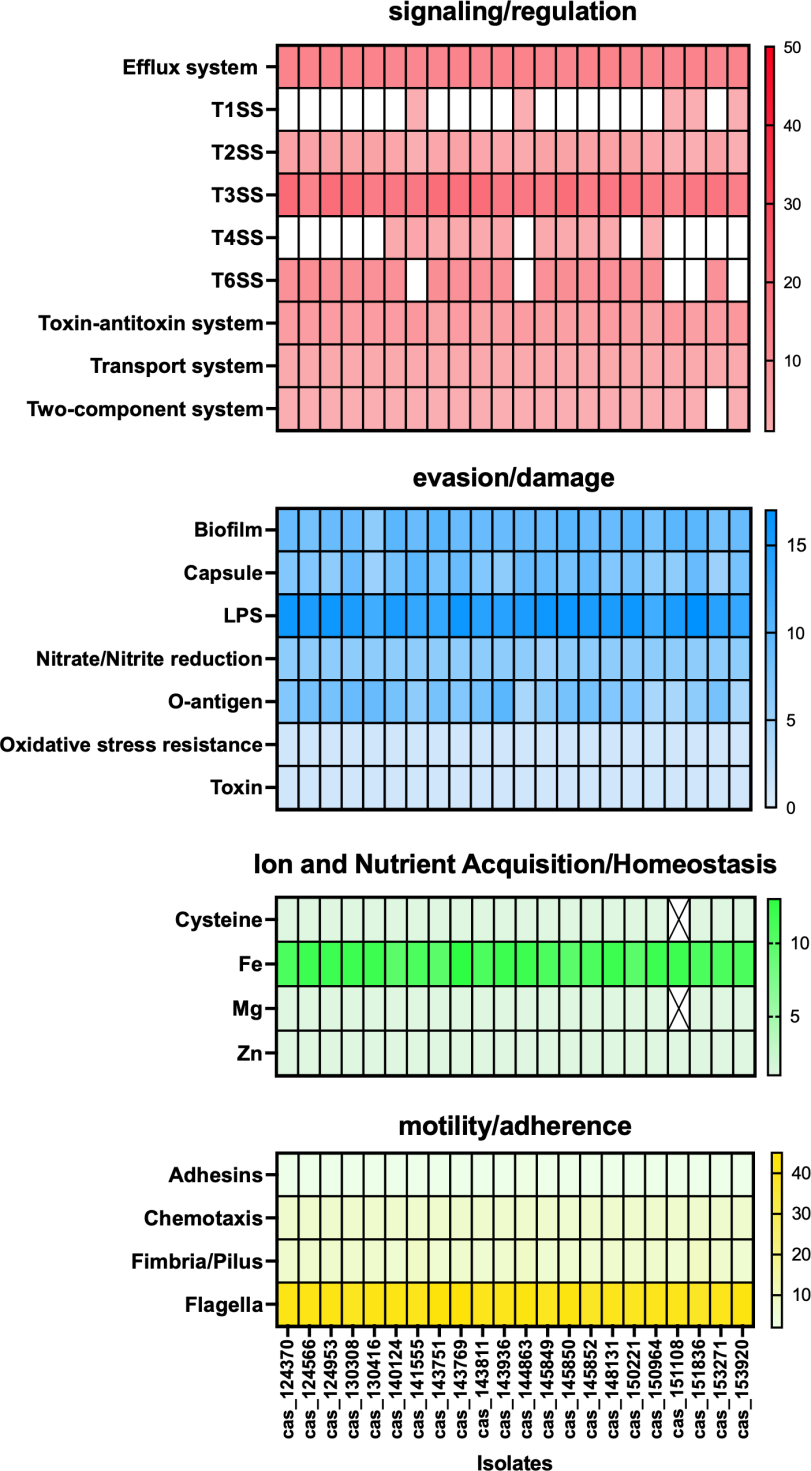
Heatmap of putative virulence genes identified in 22 *C. ginsengisoli* whole-genome sequences from clinical cases. The putative virulence factor genes were either identified by the VFDB or by manually searching through the annotated genome with selected key words. The putative virulence genes were grouped into four major functional panels: (i) signaling/regulation, (ii) evasion/damage, (iii) ion and nutrient acquisition/homeostasis, and (iv) motility/adherence. The scale of color in each cell indicates the frequency of putative virulence genes detected in the given category in each genome. Cells with an “X” mark indicate the isolate was not detected with any putative virulence gene in the corresponding category.

All identified putative virulence genes were categorized into four panels based on their potential roles in disease development (Table S6). Across isolates, the number of identified genes in each category varied: signaling/regulation (37–55 genes), evasion/damage (38–50 genes), ion and nutrient acquisition/homeostasis (13–15 genes), and motility/adherence (53–60 genes). The frequency of these genes within each panel across isolates is illustrated in a heatmap in Fig. 5.

## DISCUSSION

This study is the first to comprehensively characterize isolates of *C. ginsengisoli* from a clinical perspective. Phenotypically, we documented the colony morphology and determined the antimicrobial susceptibility profile to provide evidence-based guidance for treatment. Genomically, we identified antimicrobial resistance markers associated with phenotypic resistance, and we identified putative virulence genes that could contribute to *C. ginsengisoli* pathogenicity.

We found three different colony morphologies among the 22 isolates, underscoring the challenges in identifying *C. ginsengisoli* colonies on agar plates. While *C. ginsengisoli* colonies were small, transparent, and easily overlooked at 24 h on blood agar plates, they became larger and more distinguishable by 48 h (Fig. 1). Therefore, 48 h is the suitable time point for culture examination, especially in the presence of mixed cultures. Even with improved visualization at 48 h, reliable identification remains challenging due to two primary reasons. First, the most common colony morphology of *C. ginsengisoli* was the mucoid form (Fig. 1C), which can resemble other bacterial species such as *P. multocida* (41). This similarity means *C. ginsengisoli* can be misidentified without further molecular or proteomic confirmation. Second, the polymorphic nature of *C. ginsengisoli* colonies makes identification based solely on colony morphology insufficient, thereby further complicating the identification process (Fig. 1). Thus, more robust diagnostic tools are warranted to provide effective and specific detection of *C. ginsengisoli*.

 All *C. ginsengisoli* isolates demonstrated high MIC values for ß-lactams (Table 1; Table S3), consistent with other *Alcaligenaceae* bacteria, indicating potential intrinsic resistance of *C. ginsengisoli* (17, 18, 42–46). Therefore, based on our *in vitro* results, ß-lactams should not be used to treat *C. ginsengisoli* infections in birds. Good *in vitro* activity of aminoglycosides, sulfonamides, enrofloxacin, spectinomycin, florfenicol, and tetracyclines suggests these drugs as potential therapeutic options (Table 1). However, this contrasts with *P. multodica,* which is typically sensitive to penicillin and empirically treated with this drug class (14, 47, 48). As *C. ginsengisoli*-associated cases present similarly to *P. multocida* infections in birds (13), the divergence in the MIC profile underscores the importance of confirming the etiological agent in pasteurellosis-like infections for effective treatment. Furthermore, we identified the presence of the *aadA7* and *aph* family genes, which corresponded with high MICs for streptomycin (49, 50), while the *sul2* gene correlated with increased MICs for sulfonamides (51). On the other hand, the consistently high MIC for β-lactams suggested the presence of intrinsic resistance mechanisms for *C. ginsengisoli*. The identification of efflux pumps and ß-lactamases in all genomes could be the major mechanism of intrinsic resistance, as they are also described as intrinsic mechanisms in other members of the *Alcaligenaceae* family (17, 18, 42, 45).

We found cellular structure and putative virulence genes in *C. ginsengisoli* whole-genome sequences that may contribute to pathogenicity. We observed flagella on the cell surface under TEM (Fig. 2), a structure often associated with motility, adhesion, and biofilm formation in many other pathogens (52). Since there is no published information on *Castellaniella* virulence markers, we used other pathogens that share a similar clinical presentation (*P. multocida*) or are in the taxonomically related *Bordetella* genus to proceed with the virulence markers investigation. For instance, *P. multocida* employs superoxide dismutase genes (*sodA* and *sodC*) for immune resistance, *tonB* and *fur* for iron competition, and *ompA* and *tadD* for host cell attachment (33, 35, 53). In parallel, we identified homologous genes in *C. ginsengisoli*, including *sodB*, *tadB*, *fur*, and *ompA* (Table S6), suggesting that comparable defensive, nutrient-scavenging, and colonization strategies may be utilized. *Bordetella* delivers various toxins and colonization factors via a full complement of Type 1 to Type 6 secretion systems (T1SS to T6SS) (36, 54–56). The detection of homologous T1SS, T2SS, T3SS, T4SS, and T6SS genes in *C. ginsengisoli* implies the possession of machinery for toxin delivery or colonization. Additionally, the BvgAS two-component systems in *Bordetella* activate many virulence genes that assist in the bacteria surviving and evading the host immune response (57). Though BvgAS two-component systems were not found in the *C. ginsengisoli* genomes, there were several toxin-antitoxin homologous genes detected. In many pathogens, such as *Staphylococcus aureus, Mycobacterium tuberculosis, Escherichia coli,* and *Pseudomonas aeruginosa,* the toxin-antitoxin systems contribute to virulence via regulating cellular processes (58–60). *C. ginsengisoli* may have utilized regulatory systems to orchestrate gene expression and adjust for sudden change from the soil and water environment to animal body. Collectively, the abundant putative virulence genes identified in *C. ginsengisoli* genomes provided a foundation for understanding its mechanisms of pathogenicity.

Our analysis revealed that *C. ginsengisoli* isolates from avian lesions exhibit a closed pangenome (Fig. 4), a finding that suggests a stable and conserved genomic structure. This contrasts with a previous study that suggested environmental *Castellaniella* species have a high propensity to obtain novel genetic materials (6). The difference likely arises from the source of bacterial isolation (61, 62). While the previous study included multiple *Castellaniella* species from the environments, the current analysis focused on 23 *C. ginsengisoli* genomes, where 22 were isolated from diseased chickens and one from the environment. As the host may impose a greater fitness cost for *C. ginsengisoli* compared to a less restrictive environmental setting, the overrepresentation of clinical isolates may contribute to our closed pan-genome findings. Additionally, all the clinical *C. ginsengisoli* genomes lack plasmids, potentially restricting horizontal gene transfer and contributing to the observed closed pangenome.

While our study provided extensive characterization of *Castellaniella* genomic and phenotypic features, several limitations must be acknowledged. First, the antimicrobial susceptibility was performed solely *in vitro*; therefore, the *in vivo* activity of these drugs remained unknown. Second, the identification of putative virulence genes relied on database queries (e.g., VFDB) and searches for specific gene or gene product annotations; however, their actual functions have not been experimentally confirmed, and the current study methods have not been standardized or validated for this group of bacteria. Furthermore, although we reported the detection of specific genes, we did not verify the completeness of the corresponding operons; degenerated operons could render the predicted virulence factors unfunctional. Finally, our study may have overlooked certain virulence factors due to the limitations in the keyword selection used for the analysis.

### Conclusion

*C. ginsengisoli* may represent an emerging animal pathogen that has likely been overlooked in the past due to limited clinical and diagnostic awareness. Our findings demonstrate that reliance on colony morphology alone is insufficient for accurate identification, underscoring the need for specific diagnostic tools. The consistently elevated MIC values for β-lactams indicate intrinsic resistance within this species. Good *in vitro* activity of aminoglycosides, sulfonamides, enrofloxacin, spectinomycin, florfenicol, and tetracyclines suggests these drugs as potential therapeutic options. The genomic and electron microscopy analysis revealed the presence of flagella and a diverse repertoire of putative virulence genes, supporting the hypothesis that *C. ginsengisoli* possesses pathogenic potential. This study provides the first comprehensive phenotypic and genomic characterization of *C. ginsengisoli* clinical isolates and highlights the need for increased awareness among the medical communities regarding its distinct microbiological and genomic features.

## SUPPLEMENTAL MATERIAL

Fig. S1 spectrum.03197-25-s0001.tiff Biochemical characterization of *Castellaniella ginsengisoli* clinical isolates using the VITEK 2 GN ID System.

Table S1 spectrum.03197-25-s0002.docx *Castellaniella spp*. reference genome sequences used in this study.

Table S2 spectrum.03197-25-s0003.xlsx Keywords used for putative virulence gene search.

Table S3 spectrum.03197-25-s0004.docx ß-lactams MIC confirmation and ESBL test results for *Castellaniella ginsengisoli* using E-test and Vitek 2 GN96.

Table S4 spectrum.03197-25-s0005.xlsx Comparisons of MIC values of antimicrobials against *Castellaniella ginsengisoli* isolates using Mueller-Hinton broth with and without blood.

Table S5 spectrum.03197-25-s0006.xlsx Antimicrobial resistance genes identified by CARD, ResFinder, and AMRFinderPlus databases and by manual annotation searching.

Table S6 spectrum.03197-25-s0007.xlsx Putative virulence gene names, corresponding protein products, and assigned virulence categories.

ASM does not own the copyrights to Supplemental Material that may be linked to, or accessed through, an article. The authors have granted ASM a non-exclusive, world-wide license to publish the Supplemental Material files. Please contact the corresponding author directly for reuse.
