# Supplementary figures and images for: Phenotypic and genomic characterization of Castellaniella ginsengisoli, an emerging pathogen associated with disease in birds
Source: Microbiol Spectr. 2026 Feb 23;14(4):e03197-25. doi: 10.1128/spectrum.03197-25 (PMC13055238; doi:10.1128/spectrum.03197-25)

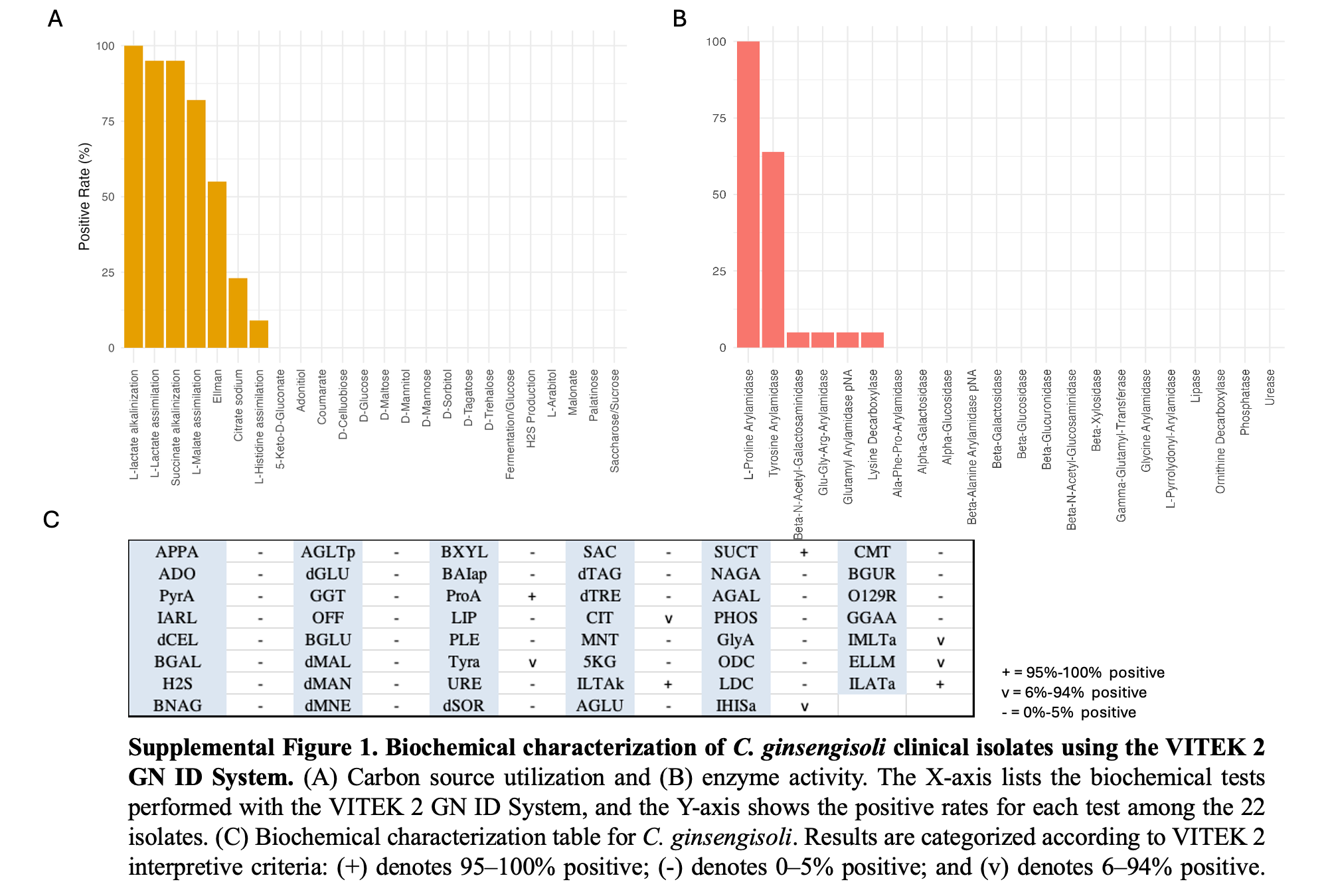

Supplement: Fig. S1 — Biochemical characterization of Castellaniella ginsengisoli clinical isolates using the VITEK 2 GN ID System. [file spectrum.03197-25-s0001.tiff]
