# Supplementary material for: Phenotypic and genomic characterization of Castellaniella ginsengisoli, an emerging pathogen associated with disease in birds
Source: Microbiol Spectr. 2026 Feb 23;14(4):e03197-25. doi: 10.1128/spectrum.03197-25 (PMC13055238; doi:10.1128/spectrum.03197-25)
Supplement: Table S1 — Castellaniella spp. reference genome sequences used in this study. [file spectrum.03197-25-s0002.docx]

**Supplemental Table 1.** ***Castellaniella* spp. reference genome sequences used in this study.**

| Sequence Name | Assembly accession number | Genome size (bp) | Submission Date | Source |
| --- | --- | --- | --- | --- |
| Castellaniella defragrans 65Phen | GCF_000612685.1 | 3,952,818 | 2/28/14 | RefSeq |
| Castellaniella caeni NBRC | GCF_001592225.1 | 3,282,217 | 3/4/16 | RefSeq |
| Castellaniella caeni | GCF_002894315.1 | 3,414,267 | 1/22/18 | RefSeq |
| Castellaniella defragrans CCUG | GCF_008801975.1 | 3,962,237 | 10/1/19 | RefSeq |
| Castellaniella defragrans DSM | GCF_014203015.1 | 3,942,929 | 8/14/20 | RefSeq |
| Castellaniella defragrans HJP | GCF_017848875.1 | 3,596,155 | 4/8/21 | RefSeq |
| Castellaniella denitrificans G21619 | GCF_027214045.1 | 3,107,010 | 12/21/22 | RefSeq |
| Castellaniella defragrans R32 | GCF_034675725.1 | 3,908,334 | 12/27/23 | RefSeq |
| Castellaniella ginsengisoli | GCF_039523235.1 | 2,912,817 | 2/23/24 | RefSeq |
| Castellaniella daejeonensis | GCF_039523555.1 | 3,122,169 | 2/23/24 | RefSeq |
| Castellaniella defragrans T2 | GCF_040394755.1 | 2,812,098 | 6/27/24 | RefSeq |
