# Supplementary material for: Phenotypic and genomic characterization of Castellaniella ginsengisoli, an emerging pathogen associated with disease in birds
Source: Microbiol Spectr. 2026 Feb 23;14(4):e03197-25. doi: 10.1128/spectrum.03197-25 (PMC13055238; doi:10.1128/spectrum.03197-25)
Supplement: Table S3 — ß-lactams MIC confirmation and ESBL test results for Castellaniella ginsengisoli using E-test and Vitek 2 GN96. [file spectrum.03197-25-s0004.docx]

**Supplemental Table 3. MICs of ß-lactams for *C. ginsengisoli* determined by E-test and Vitek 2 GN96.**

|  | Drug MIC (µg/ml) | |  | |  |  |
| --- | --- | --- | --- | --- | --- | --- |
| Isolates | Penicillin^*^ | Amoxicillin^*^ | | Amoxicillin/clavulanic acid^†^ | | |
| 124370 | >32 | >256 | | 4 | | |
| 124566 | >32 | >256 | | 8 | | |
| 124953 | >32 | >256 | | 8 | | |
| 130308 | >32 | >256 | | 8 | | |
| 130416 | >32 | >256 | | 8 | | |
| 140124 | >32 | >256 | | 8 | | |
| 141555 | >32 | >256 | | - | | |
| 143751 | >32 | >256 | | 8 | | |
| 143769 | >32 | >256 | | 8 | | |
| 143811 | >32 | >256 | | 8 | | |
| 143936 | >32 | >256 | | 8 | | |
| 144863 | >32 | >256 | | 8 | | |
| 145849 | >32 | >256 | | 8 | | |
| 145850 | >32 | >256 | | 4 | | |
| 145852 | >32 | >256 | | 4 | | |
| 148131 | >32 | >256 | | 8 | | |
| 150221 | >32 | >256 | | 8 | | |
| 150964 | >32 | >256 | | 8 | | |
| 151108 | >32 | >256 | | 4 | | |
| 151836 | >32 | >256 | | - | | |
| 153271 | >32 | >256 | | 8 | | |
| 153920 | >32 | >256 | | 8 | | |

^*^ MIC determined by E-test

^†^ MIC determined by Vitek 2 GN96 card

MICs = minimum inhibitory concentrations; E-test = Epsilometer testing.
